# Modeling the Impact of Ergonomic Interventions and Occupational Factors on Work-Related Musculoskeletal Disorders in the Neck of Office Workers with Machine Learning Methods

**DOI:** 10.34172/jrhs.2024.158

**Published:** 2024-07-31

**Authors:** Mohammad Sadegh Sohrabi, Hassan Khotanlou, Rashid Heidarimoghadam, Iraj Mohammadfam, Mohammad Babamiri, Ali Reza Soltanian

**Affiliations:** ^1^Center of Excellence for Occupational Health, Occupational Health and Safety Research Center, Hamadan University of Medical Sciences, Hamadan, Iran; ^2^Department of Computer Engineering, Bu-Ali Sina University, Hamedan, Iran; ^3^Department of Ergonomics, School of Public Health, Hamadan University of Medical Sciences, Hamadan, Iran; ^4^Research Center for Health Sciences, Hamadan University of Medical Sciences, Hamadan, Iran; ^5^Department of Ergonomics, Health in Emergency and Disaster Research Center, University of Social Welfare and Rehabilitation Sciences, Tehran, Iran; ^6^Modeling of Noncommunicable Diseases Research Center, Hamadan University of Medical Sciences, Hamadan, Iran; ^7^Department of Biostatistics, School of Public Health, Hamadan University of Medical Sciences, Hamadan, Iran

**Keywords:** Ergonomics, Model, Machine learning, Support vector machine

## Abstract

**Background:** Modeling with methods based on machine learning (ML) and artificial intelligence can help understand the complex relationships between ergonomic risk factors and employee health. The aim of this study was to use ML methods to estimate the effect of individual factors, ergonomic interventions, quality of work life (QWL), and productivity on work-related musculoskeletal disorders (WMSDs) in the neck area of office workers.

**Study Design:** A quasi-randomized control trial.

**Methods:** To measure the impact of interventions, modeling with the ML method was performed on the data of a quasi-randomized control trial. The data included the information of 311 office workers (aged 32.04±5.34). Method neighborhood component analysis (NCA) was used to measure the effect of factors affecting WMSDs, and then support vector machines (SVMs) and decision tree algorithms were utilized to classify the decrease or increase of disorders.

**Results:** Three classified models were designed according to the follow-up times of the field study, with accuracies of 86.5%, 80.3%, and 69%, respectively. These models could estimate most influencer factors with acceptable sensitivity. The main factors included age, body mass index, interventions, QWL, some subscales, and several psychological factors. Models predicted that relative absenteeism and presenteeism were not related to the outputs.

**Conclusion:** In this study, the focus was on disorders in the neck, and the obtained models revealed that individual and management interventions can be the main factors in reducing WMSDs in the neck. Modeling with ML methods can create a new understanding of the relationships between variables affecting WMSDs.

## Background

 Today, in many sciences, modeling is used to understand the current situation or predict the future of phenomena. In this manner, by using various computer and calculation methods, the complex relationships between variables are determined, and the future behavior of the phenomenon can be predicted with an acceptable level of error.^1^ In ergonomics, the use of several methods such as conceptual modeling, field-scale modeling, laboratory-scale modeling, mathematical modeling, statistical modeling, or computational modeling leads to a better understanding of phenomena related to humans and work, health, or productivity.^2-4^ Some modeling methods are utilized to create digital humans and estimate human characteristics in the simulation environment, which is called digital human modeling.^5^ Some other modeling methods employ artificial intelligence and machine learning (ML) capabilities to understand the complex relationships between human risk factors such as work-related musculoskeletal disorders (WMSDs).^6^ In these methods, various statistical methods have been invented and developed to explain the relationship between the outcome (dependent variable) and the factors of creation (independent variables), in situations where the number of explanatory variables is large and they have extensive diversity.^5^ These methods are highly diverse according to the nature of the dependent variable and how the explanatory variables are. In classical statistics, it is common to use common regression models for such situations. However, these models have a complex structure and often have strict preconditions, such as establishing a normal distribution and homogeneity of variances, and the like, and the failure to establish these preconditions nearly limits the use of these models.^1^ Therefore, there is a need to invent new methods that, in addition to overcoming the above conditions, can achieve acceptable results with high speed and fewer calculations.^3^

 Most of the studies that have so far been conducted in the field of ergonomics have used regression models to estimate the prevalence of WMSDs (including repetition, severity, and interference with work), assuming a linear relationship between musculoskeletal disorders and effective factors. This assumption can lead to an incorrect estimate of factors affecting the prevalence of WMSDs because there may be complex, non-linear relationships between factors affecting the prevalence of WMSDs.^2,7^ In this regard, methods called decision trees (DT) and tree models have been invented and developed that can cover a significant part of these needs. DT is one of the most famous classification techniques utilized in the data mining process. The method of displaying the DT is that it summarizes the classification procedure by presenting a tree. DTs are employed to predict the membership of objects in different categories.^1,8^ The flexibility of this technique has made it more widely used among attractive data mining methods.^4^ Another method that uses ML to classify data is called support vector machines (SVMs), applying various data classification algorithms and functions to recognize and differentiate complex patterns in data and classify them.^9^

 Among these newer studies that have used ML methods in data analysis and modeling, there are examples in the analysis of factors causing WMSDs and the relationships between these factors,^4,6,10,11^ biomechanical studies of posture and manual handing,^12-15^ fatigue modeling,^16^ burnout modeling,^17^ measurement and analysis of mental workload and electroencephalography.^9,18-20^ The purpose of the present study is to use ML methods to estimate the effect of individual factors, ergonomic interventions, quality of work life (QWL), and productivity on WMSDs in the neck area of office workers in small and medium enterprises.

## Methods

**Figure 1:**
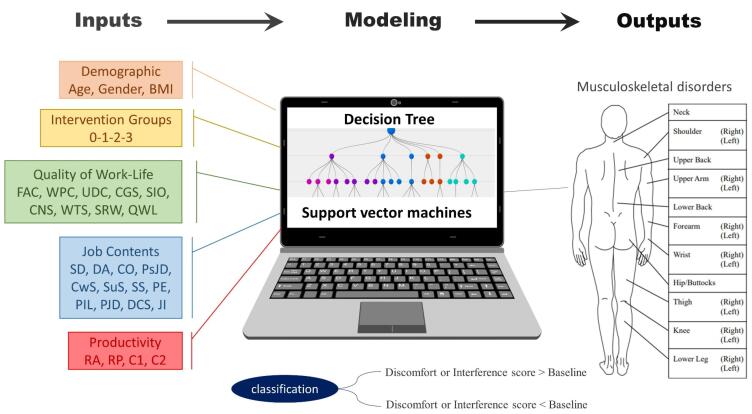
The main framework of the model

 The data required for modeling in the present study were collected from the results of a quasi-randomized control trial conducted in 2019–2020.^21^ In that field study, the participants were randomly divided into four parallel groups, three of which were subjected to intervention, and the fourth group was utilized without intervention and only for control. The intervention groups included individual ergonomic training, management training and work changes, and both individual and management training. The study population included all white-collar employees of knowledge-based companies located in Isfahan Science and Technology Town, Iran. The participants included 311 white-collar workers (112 women and 199 men), with a mean age of 32.04 and a standard deviation of 5.34 years. The mean body mass index (BMI) was 24.53 ± 3.35 kg/m^2^. The obtained data shown in the previous paper^22^ were used for modeling with the ML method. To determine the relationships between the measured variables and the relationship pattern between these variables, the collected data were divided into two categories of input variables. They included demographic information of the participants, components of the QWL, measured components of job content based on the demand-control-support model, variables of perceived productivity, three types of interventions performed during the field study, and the output of the model, including WMSDs in the neck. These variables were available at baseline, one, three, and six months after the implementation of the intervention. The classification was considered in two cases of the increase or decrease of WMSDs compared to the baseline. The main framework of the model is shown in Figure 1.

 In the Cornell Musculoskeletal Discomfort Questionnaires, three components are measured at the same time, namely, frequency (including five options: never, 1–2 times a week, 3–4 times a week, every day, and several times a day), discomfort (low, moderate, and very high), and interference with work (not at all, low, and high). The inventor of this tool, Dr. Alan Hedge, suggests various methods for weighing the obtained answers,^23^ which include counting the signs of each person, summing the signs for each person, and weighing the answers (which was used in the field study). In addition, the score of the three parts is repetition, discomfort, and interference with work.^24^ For modeling, first, the outputs from the final score (frequency × discomfort × interference) of the components of the final score, such as the final score of WMSDs in the neck, were selected. However, due to the unbalanced distribution of this variable (a number between 0 and 90), the modeling was not performed with proper accuracy. Next, the frequency variable was utilized as the output. In this situation, due to the exponential value of this variable (0, 1.5, 3.5, 5, and 10), proper accuracy was not obtained in the modeling. However, in the proposed scoring for this tool, Hedge himself also states that the reason for choosing this scale for repeating or the overall effect of the symptoms of WMSDs is to give double importance to the situations that are complementary to the pain and discomfort. He suffers from and deals with this issue repeatedly during the week. However, in the scoring guide, Hedge suggests that linear or counting methods can also be employed for numerical scoring.^24^ Finally, occurrence and non-occurrence modes were applied in the model designed for the frequency of WMSDs and their severity. Therefore, the explanations and testing of various variables were used in the outputs of the model from the numerical value of the discomfort score and interference with work. Further, according to the follow-up stages of the field study, the value of this variable was considered in each of the follow-up stages of one, three, and six months compared to the value of the pre-intervention stage (the baseline). Considering the large number of features that could affect the output of the model, first, neighborhood component analysis (NCA) was utilized to identify the most important features. NCA was employed to select the influential input variables in predicting the model outputs and weighting the impact of these variables.

 The calculation was such that in the model, numerical value changes, compared to the baseline stage, were considered to increase or decrease in two classes, and two-state classification was implemented to increase the accuracy of the modeling. Algorithms fine tree, bagged tree, medium tree, coarse tree, RUSBoosted tree in binary form, and SVMs, including quadratic SVM, linear SVM, Gaussian SVM, medium Gaussian SVM, and subspace discriminant of decision tree were used in machine learning by programming in MATLAB 2020 software.

 In the current study, the output of the model was determined in two binary classes, namely, the decrease or decrease of musculoskeletal disorders, and they were nominal with high deviation. Furthermore, the number of data and outlier data were not balanced in the two designated classes, so DT and SVM were used for modeling. Gini’s diversity index, One-vs-One, and discriminant techniques were used in this regard. SVM is a type of classification algorithm that separates data points using a line. This dividing line is chosen to be the closest line between the two groups. A DT algorithm also classifies data for several sets based on some selected characteristics (independent variables) of a population. The classification was defined for the model in the two cases of increasing the score of discomfort or interfering with the work compared to the initial evaluation values and decreasing it. Moreover, to determine the accuracy and acceptable validity of the model in each of the body parts, outputs that had at least 25 non-zero data and the number of balanced class elements were considered for modeling at each stage of the follow-ups. Therefore, modeling was not performed due to the presence of zero data reported by the participants in many body parts in the first, third- and sixth-month follow-ups.

 Additionally, the validation method of the 5-fold cross-validation model is taken into consideration. About 80% of the data were employed for machine training, and the remaining 20% was used for model testing. This segmentation was performed five times with different parts of the data. This method determines the extent to which the results of a statistical analysis on a data set are generalizable and independent of the data utilized to train the model. Performance measurement parameters, including accuracy, the area under the curve, and the receiver operating characteristic curve, were applied to check the performance of the obtained models.

## Results

**Figure 2:**
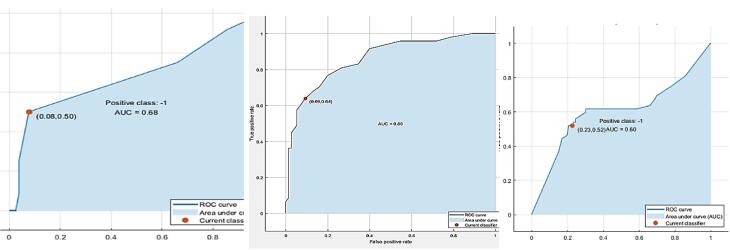
ROC curves: one-month follow-up model (left), three-month follow-up model (middle), and six-month follow-up model (right). *Note*. ROC curve: Receiver operating characteristic curve

 Due to the nature of the data subjectively reported MSDs in all body parts in the three follow-up times, the outcome values were zero or were not reported, and acceptable models were obtained for all three follow-up times only in the neck area. After analyzing the data using ML algorithms, the top three models were selected based on 1-, 3-, and 6-month follow-ups. The characteristics of these models are presented in Table 1 and Figure 2. The accuracy of the designed model was estimated at 86.5%, 80.3%, and 69% in the first-, third-, and sixth-month follow-up models, respectively. Despite the 20% decrease in model accuracy until the sixth month, an acceptable rate of accuracy was observed in all three models. The sensitivity of these three models was determined to be 0.68, 0.86, and 0.6, respectively. Additionally, based on NCA analysis, the main influential inputs for predicting discomfort or interference with work in the neck area were determined, including age, BMI, performed interventions, salary level, working environment conditions, general atmosphere of working life, and ability to use individual skills. The details of these models in the mode of considering all the features and in the mode of considering only the important features are included in Supplementary file 1.

**Table 1:** The best-fitted SVM-based model based on prioritized factors for three follow-up times

**Variables**	**One-month follow-up**	**Three-month follow-up**	**Six-month follow-up**
Accuracy	86.5%	80.3%	69.0%
Sensitivity (AUC)	68.2%	86.1%	60.5%
Model type	Fine tree	Bagged tree	Medium tree
Most influencer factors	Age, BMI, intervention, UDC, SIO, CNS, WTS, SD, CO, PsJD, SuS, and DCS	Age, BMI, intervention, FAC, WPC, CNS, WTS, SRW, SD, DA, PsJD, SuS, and PIL	Age, BMI, intervention, FAC, WPC, UDC, SIO, CNS, SRW, QWL, DA, CO, PsJD, PE, PJD, and JI

*Note*. SVM: Support vector machines; BMI: Body mass index; FAC: Fair and appropriate compensation; WPC: Workplace conditions; UDC: Use and development of capacities; CGS: Chance of growth and security; SIO: Social integration in the organization; CNS: Constitutionalism; WTS: Work and the total space of life; SRW: Social relevance of the work in the life; QWL: Quality of work life; SD: Skill discretion; DA: Decision authority; CO: Control; PsJD: Psychological job demands; SuS: Supervisor support; PE: Physical exertion; PIL: Physical isometric load; PJD: Physical job demands; DCS: Demand control support status; JI: Job insecurity; ROC: Receiver operating characteristic; AUC: Area under curve.

**Figure 3:**
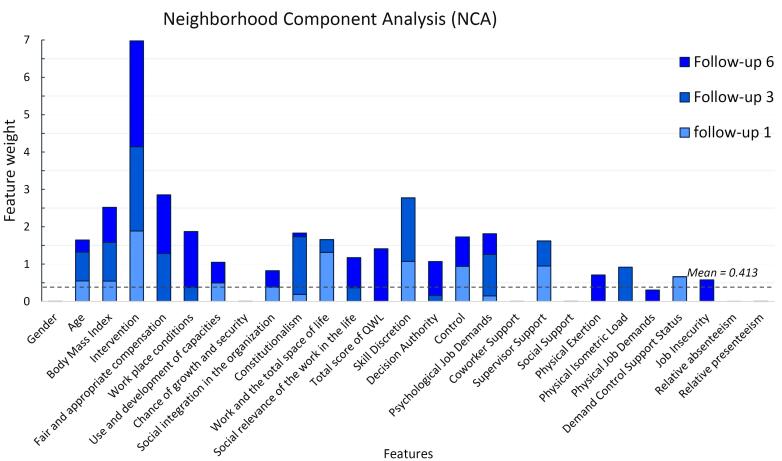
Neighborhood component analysis for estimating outputs

 The sensitivity of the models based on the area under the curve was obtained as 0.68, 0.86, and 0.60, respectively. Table 1 provides the receiver operating characteristic curve for all three models. In general, based on the results of three models designed to determine the input variables, the most influential factors in predicting the increase or decrease of discomfort and work interference in the neck were age, BMI, type of implemented intervention, fair and appropriate compensation, workplace conditions, use and development of capacities, and social integration in the organization. The other influential factors included constitutionalism, work and the total space of life, QWL, skill discretion, decision authority, control, psychological job demands, supervisor support, physical exertion, physical isometric load, physical job demands, demand control support status, and job insecurity. More specifically, intervention type, fair and appropriate compensation, skill discretion, and BMI had the greatest effect, respectively, while physical job demands, job insecurity, demand control support status, and physical exertion had the least effect on predicting the output variable of the model. The details of the weighting of these variables in the models are depicted in the NCA diagram in Figure 3, with three follow-up times.

## Discussion

 With DT and SVM methods, appropriate models were designed with acceptable accuracy and sensitivity. These models estimate the impact of input variables in five categories of demographic variables, type of implemented interventions, QWL and its indicators, job contents, and perceived productivity components on WMSDs in the neck and have an acceptable classification power in predicting the increase or decrease of model outputs based on weighted effective variables. Due to the type of tool used to collect information on WMSDs and participants’ data in other body parts, it was impossible to model with acceptable accuracy and precision, and only the neck was designed for all three measured follow-ups. The first- and third-month follow-up models had the highest accuracy and highest sensitivity, respectively. Unfortunately, a handful of studies have modeled the impact of ergonomic interventions on WMSDs in the neck and upper limbs with ML methods; thus, in this section of the discussion, the results will be compared with the results of the repeated measures ANOVA (published in the authors’ previous paper ^22^) and modeling with the ML method (results of this paper) and some similar studies.

 In repeated measures of ANOVA analysis,^22^ only BMI was an effective variable in predicting changes in the prevalence of WMSDs in the neck, but in modeling with the ML method, age was also identified as an effective factor in predicting changes in the severity of WMSDs. In previous consistent studies, both age and BMI were effective on WMSDs.^6,7,25^ In both analyses, the implemented interventions were strong factors for controlling and managing WMSDs, which is in line with the results of other similar studies,^4,7,25,26^ demonstrating the great impact of ergonomic interventions on controlling WMSDs.

 In the subscales of the QWL, fair and appropriate compensation, workplace conditions, use and development of capacities, social integration in the organization, constitutionalism and work, and the total space of life were effective in estimating WMSDs. The designed models did not consider the high weight for the effect of QWL, but fair and appropriate compensation and workplace conditions were determined to be more effective, especially in the sixth-month follow-up, which has taken place due to economic inflation in 2020 in Iran, the corona virus disease and job restrictions imposed due to the epidemic in work environments.^27-29^

 In the analysis of the effect of the input variables of the job content questionnaire, which is based on the job-demand-control-support model, on the changes of musculoskeletal disorders in the neck, skill discretion, decision authority, control, psychological job demands, supervisor support, physical exertion, physical isometric load, physical job demands, demand control, support status, and job insecurity were effective. The greatest impact of the input component was related to skill discretion, psychological job demands, and control, respectively. This should be due to the type of work activities in the studied companies, where people have different roles and responsibilities at work, and the main form of these tasks is psychological, which is in line with the results of other studies,^27,28,30^ suggesting the mediating effect of psychosocial stressor factors for MSDs. In addition, the effect of these components was confirmed in the analysis of the repeated measures ANOVA model.^22^

 In modeling with the ML method, support variables were either not included in the model or were not recognized as strong factors; however, in the ANOVA model, the support of colleagues and supervisors was determined to be effective in reducing WMSDs.^22^ The results revealed that the social aspects of work, such as support, have less impact on MSDs than the physical aspects of work, which can be due to the greater impact of biomechanical factors on MSDs. The social aspects of work can be more effective on higher burnout and lower work engagement.^30^ DCS model status includes four statuses. “Active/passive/low-strain/high-strain” was not determined to be a strong component for predicting MSDs. The findings of Yu et al also demonstrated that the DCS model alone cannot be a good predictor for WMSDs, and there is a need for other combined models ^28^. However, the results of a previous study indicated that the three main factors of the Karasek model, namely, demands, control, and support, were effective in reducing the incidence of MSDs.^22^

 According to the NCA, relative absenteeism and relative presenteeism were not effective in predicting WMSDs in the neck area and were not included in the models. Nonetheless, in the ANOVA model, relative absenteeism is a predictor of WMSDs, but relative presenteeism, similar to the results of this modeling, has not been determined to be an effective factor in changes in WMSDs in the neck area.^22^

 These differences can be considered due to the limitations of choosing the type of variables in the ANOVA model. Because in these statistical methods, some nominal variables cannot be included in the analysis. Further, the use of these methods for detecting relationships between causal and effectual variables has other structural limitations. However, in the ML method, the artificial intelligence algorithm created in repeated and long cycles examines the relationships between the input and output variables, discovers non-linear relationships between these elements through trial and error during the learning process, and confirms it in another process.^3,9,10^. Furthermore, the accuracy of the models obtained by the ML method in the present study (between 69% and 86.5%) has an acceptable superiority over those reported in previous studies.^4,16^ However, in the study by Hanumegowda and Gnanasekaran,^11^ although they used nominal data on musculoskeletal disorders, they reported 100% accuracy of the final model presented by the DT method and random forest algorithms, the most important reason being the difference in the number and dispersion of their data. Conversely, compared to other studies of ergonomic modeling with ML methods that modeled with continuous data such as electrocardiogram, electroencephalogram, or electromyogram,^9,18,20^ kinetic or kinematic data of posture detection,^12-15^ or quantitative data for manual handling risk assessment,^31^ the accuracy of our models was less, which can be due to the difference in the nature of the data used for modeling or the number of features affecting the model outputs.

**Highlights**

- The application of ML techniques, such as SVMs and decision trees, has enabled the identification of the impact of various personal and occupational factors on the occurrence and prevalence of musculoskeletal disorders.
- Age, BMI, interventions, QWL, and psychological factors were included as the main effect characteristics in the models.
- Ergonomic interventions at the individual and management levels can be the main factors in reducing neck discomfort and interference with work.

## Conclusion

 Modeling with ML methods can create a newer understanding of the relationships between variables affecting WMSDs with acceptable accuracy and sensitivity. The models implemented with the DT algorithm with the fine tree method were more accurate than bagged, boosted, or medium trees. In this study, the focus was on disorders in the neck, and the obtained models showed that individual and management interventions can be the main factors in reducing discomfort and interference with work in the neck. Moreover, individual factors such as age and BMI are predictors of MSDs, along with some factors that create the quality of working life. According to the designed models, psychological factors are as effective as physical factors.

 Several strengths and limitations of the study warrant consideration. First, due to the discreteness of the data of WMSDs, the possibility of modeling was provided only for the neck region, which was more prevalent among the results, and no suitable model was obtained for other body parts. Second, the field data used in this study were related to office workers in small and medium enterprises, which limits the generalizability of the results to other work communities. Third, modeling with the ML method has a variety and multiplicity of tools and methods, which, compared to previous methods such as ANOVA, can have its own strengths and weaknesses that require the attention of researchers. Eventually, the ML method in data analysis can create a newer picture of the relationships between variables, which creates a wider understanding of the factors and risk factors of ergonomics in work environments.

## Acknowledgments

 The authors would like to thank all workers, employees, and managers participating in the study.

## Authors’ Contribution

**Conceptualization:** Mohammad Sadegh Sohrabi.

**Data curation:** Mohammad Sadegh Sohrabi.

**Formal analysis:** Ali Reza Soltanian.

**Methodology:** Hassan Khotanlou.

**Supervision:** Rashid Heidarimoghadam, Iraj Mohammadfam, Mohammad Babamiri.

**Visualization:** Mohammad Sadegh Sohrabi.

**Writing–original draft:** Mohammad Sadegh Sohrabi, Mohammad Babamiri.

**Writing–review & editing:** Mohammad Sadegh Sohrabi, Hassan Khotanlou.

## Competing Interests

 The authors declare that they have no competing interests.

## Ethical Approval

 This study was approved by the Ethics Committee of Hamadan University of Medical Sciences under registration number IR.UMSHA.REC.1397.688.

## Funding

 The study was financially supported by the Vice-Chancellor for Research and Technology, Hamadan University of Medical Sciences (IR.UMSHA.REC.1397.688).

## Supplementary Files


**Supplementary file 1.** Modeling details
