# Supplementary material for: Modeling the Impact of Ergonomic Interventions and Occupational Factors on Work-Related Musculoskeletal Disorders in the Neck of Office Workers with Machine Learning Methods
Source: J Res Health Sci. 2024 Jul 31;24(3):e00623. doi: 10.34172/jrhs.2024.158 (PMC11380738; doi:10.34172/jrhs.2024.158)
Supplement: Supplementary file 1 — Modeling details [file jrhs-24-e00623-s001.pdf]

doi:10.34172/jrhs.2024.158
JRHS 2024; 24(3):e00623

Supplementary file 1: Details of machine learning models

Estimating the impact of input variables on reducing or increasing musculoskeletal disorders in the neck, the features used in this model are in order of number

Gender	1	Work and the total space of life (WTS)	11	Physical Exertion (PE)	21
Age (yr)	2	Social relevance of the work in the life (SRW)	12	Physical isometric load (PIL)	22
Body mass index (BMI)	3	Total score of QWL (QWL)	13	Physical job demands (PJD)	23
Intervention	4	Skill discretion (SD)	14	Demand control support status (DCS)	24
Fair and appropriate compensation (FAC)	5	Decision authority (DA)	15	Job Insecurity (JI)	25
Work place conditions (WPC)	6	Control (CO)	16	Relative absenteeism (RA)	26
Use and development of capacities (UDC)	7	Psychological job demands (PsJD)	17	Relative presenteeism (RP)	27
Chance of growth and security (CGS)	8	Coworker support (CwS)	18	Combining absenteeism and presenteeism (C1)	28
Chance of growth and security (SIO)	9	Supervisor support (SuS)	19	Lost performance (C2)	29
Constitutionalism (CNS)	10	Social support (SS)	20		

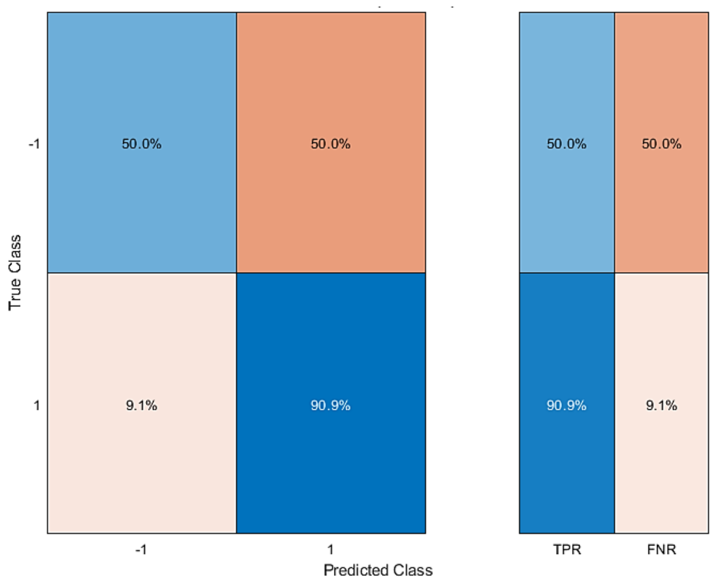

Results

Accuracy	85.4%
Total misclassification cost	13
Prediction speed	~970 obs/sec
Training time	4.5216 sec

Model Type

Preset: Fine Tree
Maximum number of splits: 100
Split criterion: Gini's diversity index
Surrogate decision splits: Off

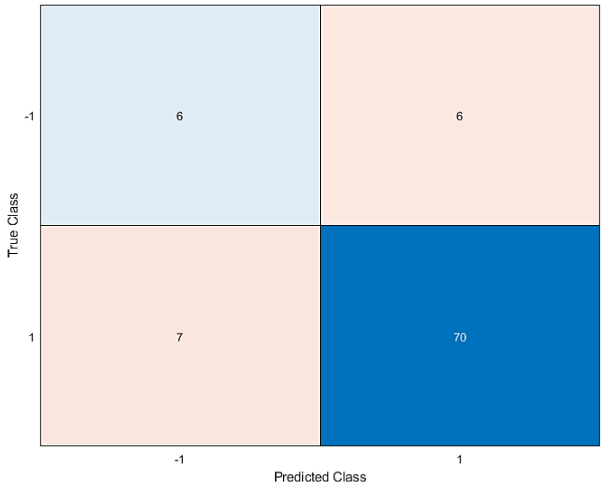

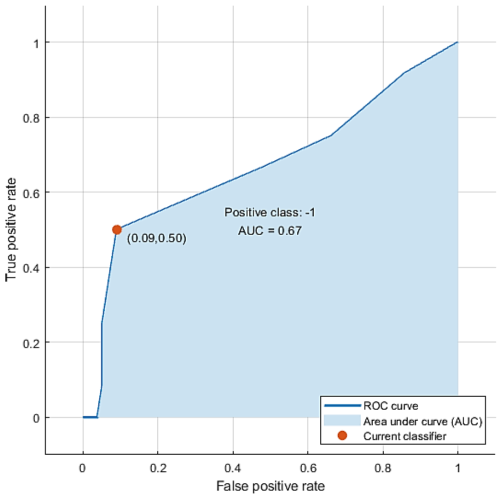

Model 1.1. Output for reduction or increase (binary) of musculoskeletal disorders in the neck with all features, Follow-up 1 month. Fine tree model.

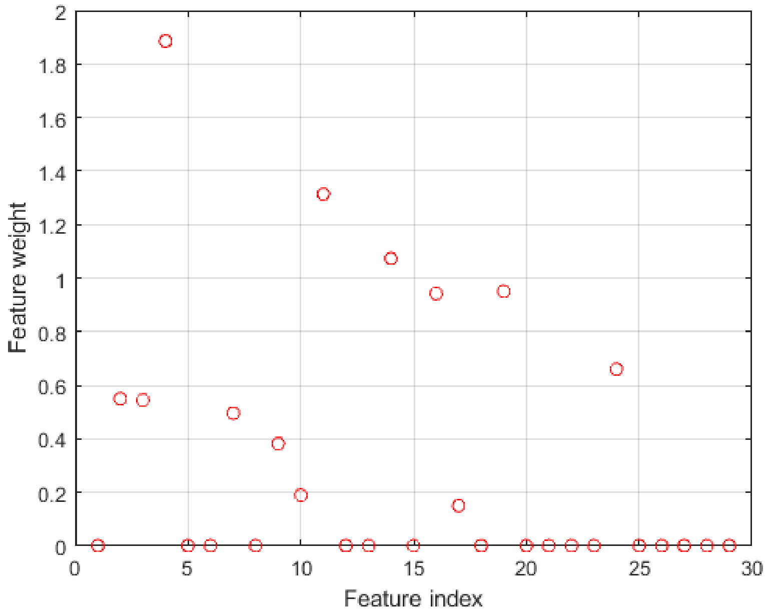

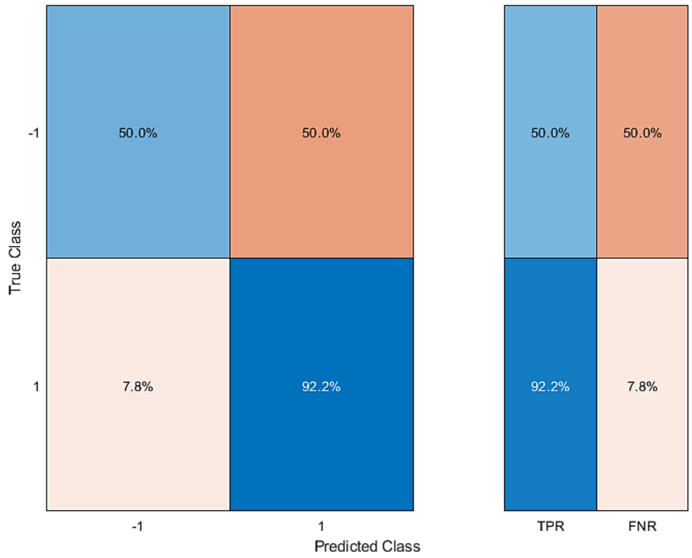

Results

Accuracy	86.5%
Total misclassification cost	12
Prediction speed	~3000 obs/sec
Training time	1.7912 sec

Model Type
Preset: Fine Tree
Maximum number of splits: 100
Split criterion: Gini's diversity index
Surrogate decision splits: Off

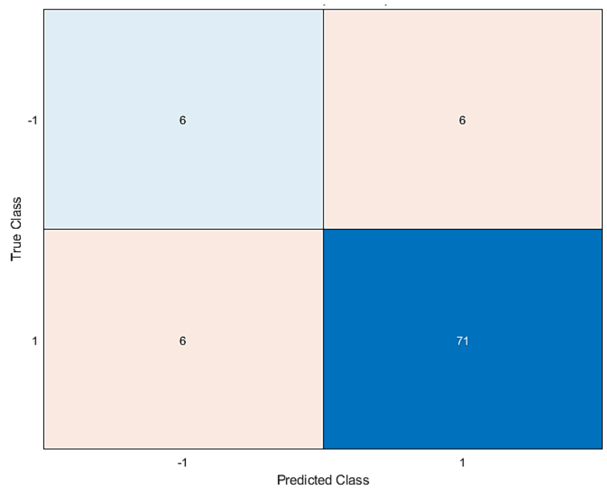

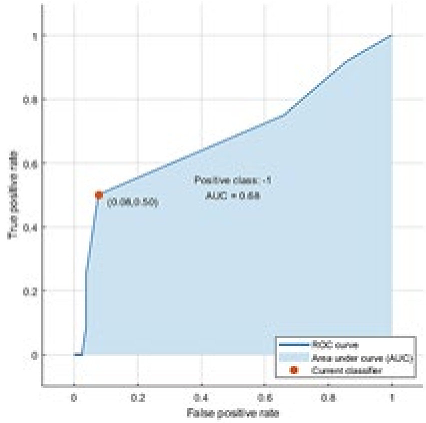

Model 1.2. Output for reduction or increase (binary) of musculoskeletal disorders in the neck <u>with selected features</u> According to the diagram below, Follow-up 1 month. Fine tree model.

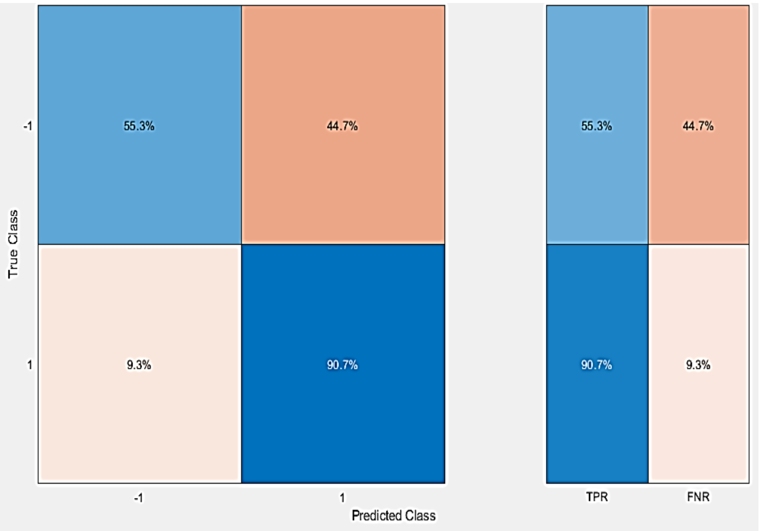

Results

Accuracy	77.0%
Total misclassification cost	28
Prediction speed	~630 obs/sec
Training time	5.6578 sec

Model Type

Preset: Bagged Trees
Ensemble method: Bag
Learner type: Decision tree
Maximum number of splits: 121
Number of learners: 30

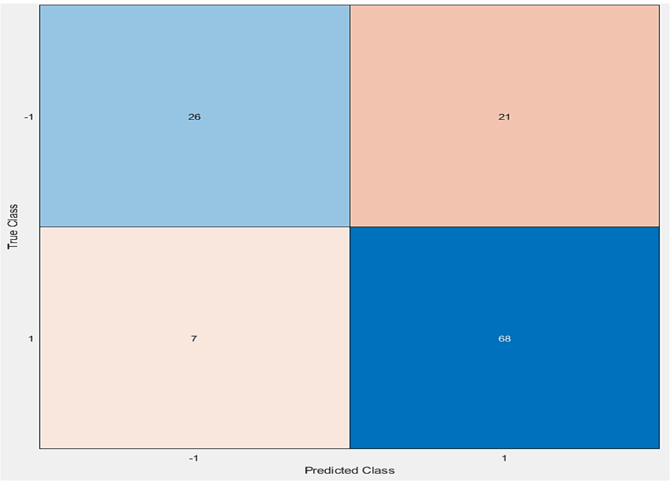

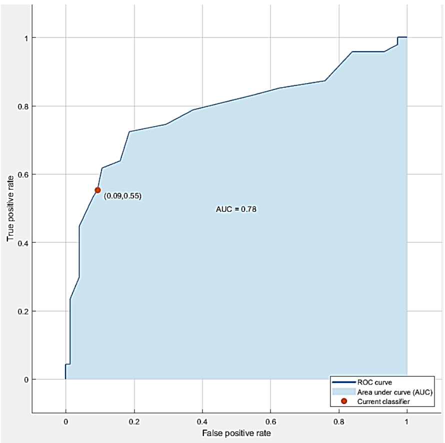

Model 2.1. Output for reduction or increase (binary) of musculoskeletal disorders in the neck with all features, Follow-up 3 months. Bagged trees model.

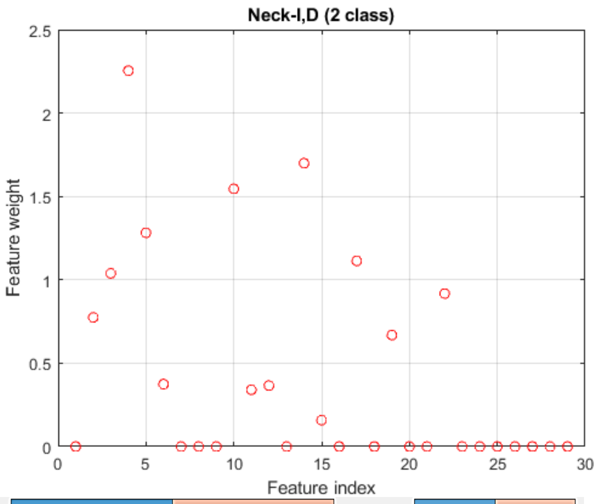

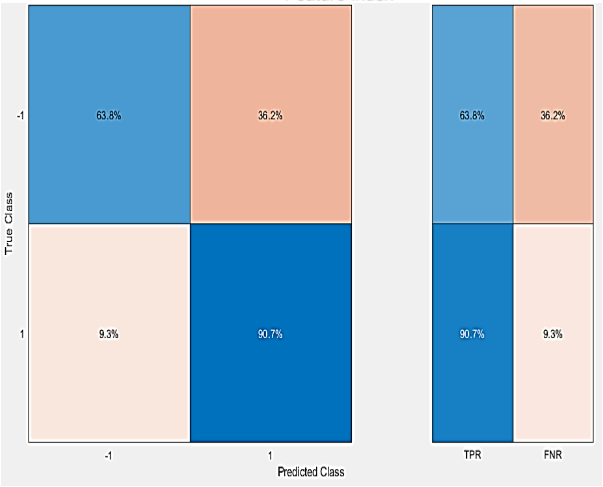

Results

Accuracy	80.3%
Total misclassification cost	24
Prediction speed	~500 obs/sec
Training time	4.2251 sec

Model Type

Preset: Bagged Trees
Ensemble method: Bag
Learner type: Decision tree
Maximum number of splits: 121
Number of learners: 30

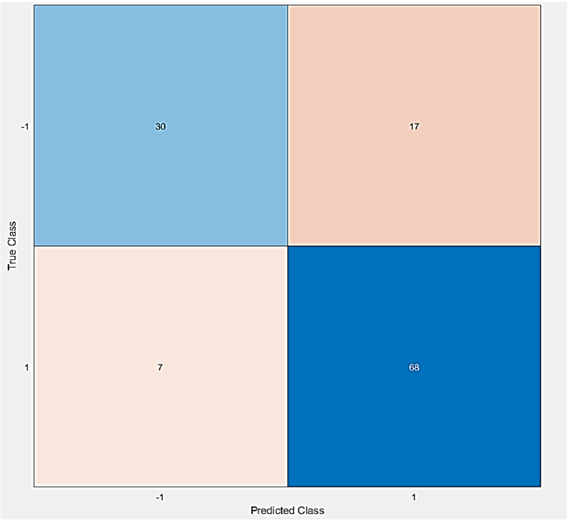

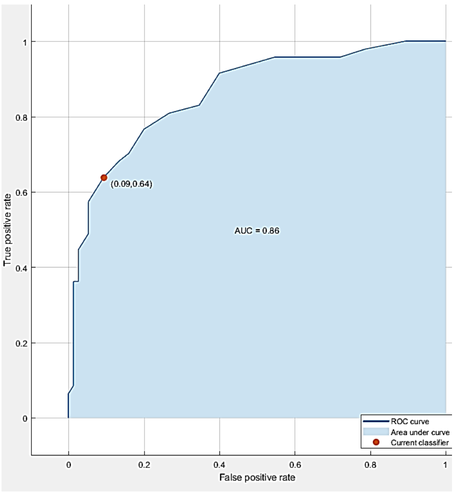

Model 2.2. Output for reduction or increase (binary) of musculoskeletal disorders in the neck with selected features According to the diagram below, Follow-up 6 month. Medium tree model.

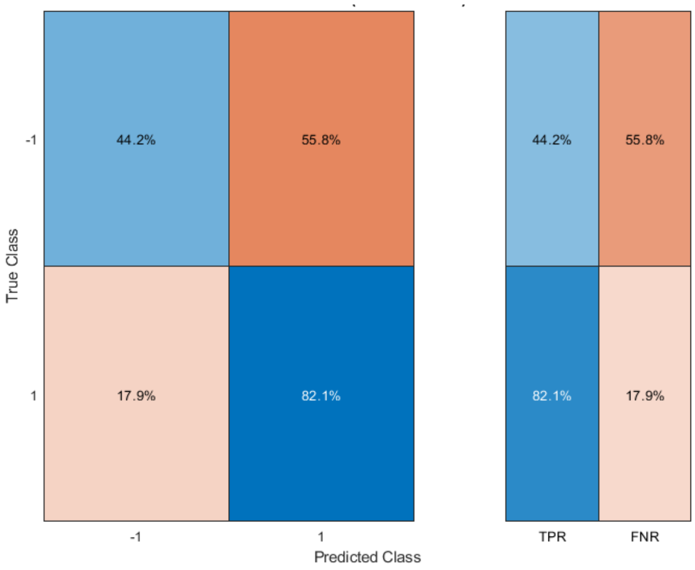

Results

Accuracy	69.6%
Total misclassification cost	48
Prediction speed	~1200 obs/sec
Training time	2.4425 sec

Model Type

Preset: Boosted Trees
Ensemble method: AdaBoost
Learner type: Decision tree
Maximum number of splits: 20
Number of learners: 30
Learning rate: 0.1

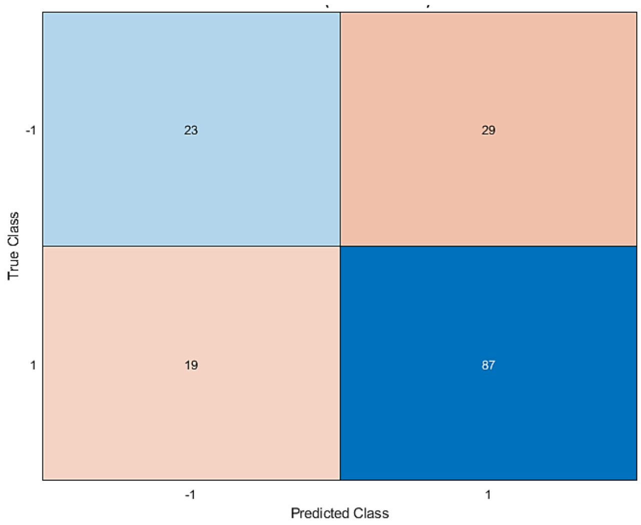

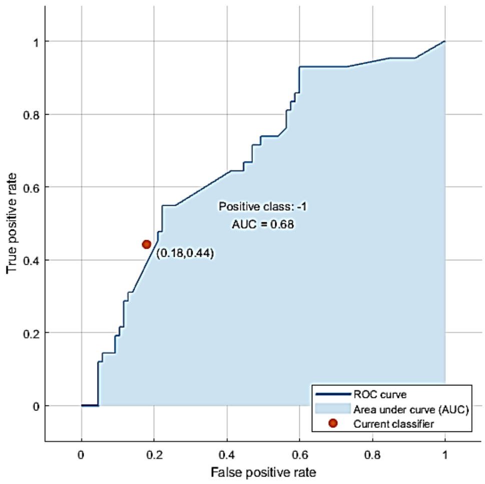

Model 3.1. Output for reduction or increase (binary) of musculoskeletal disorders in the neck <u>with all features</u>, Follow-up 6 months. Boosted trees model.

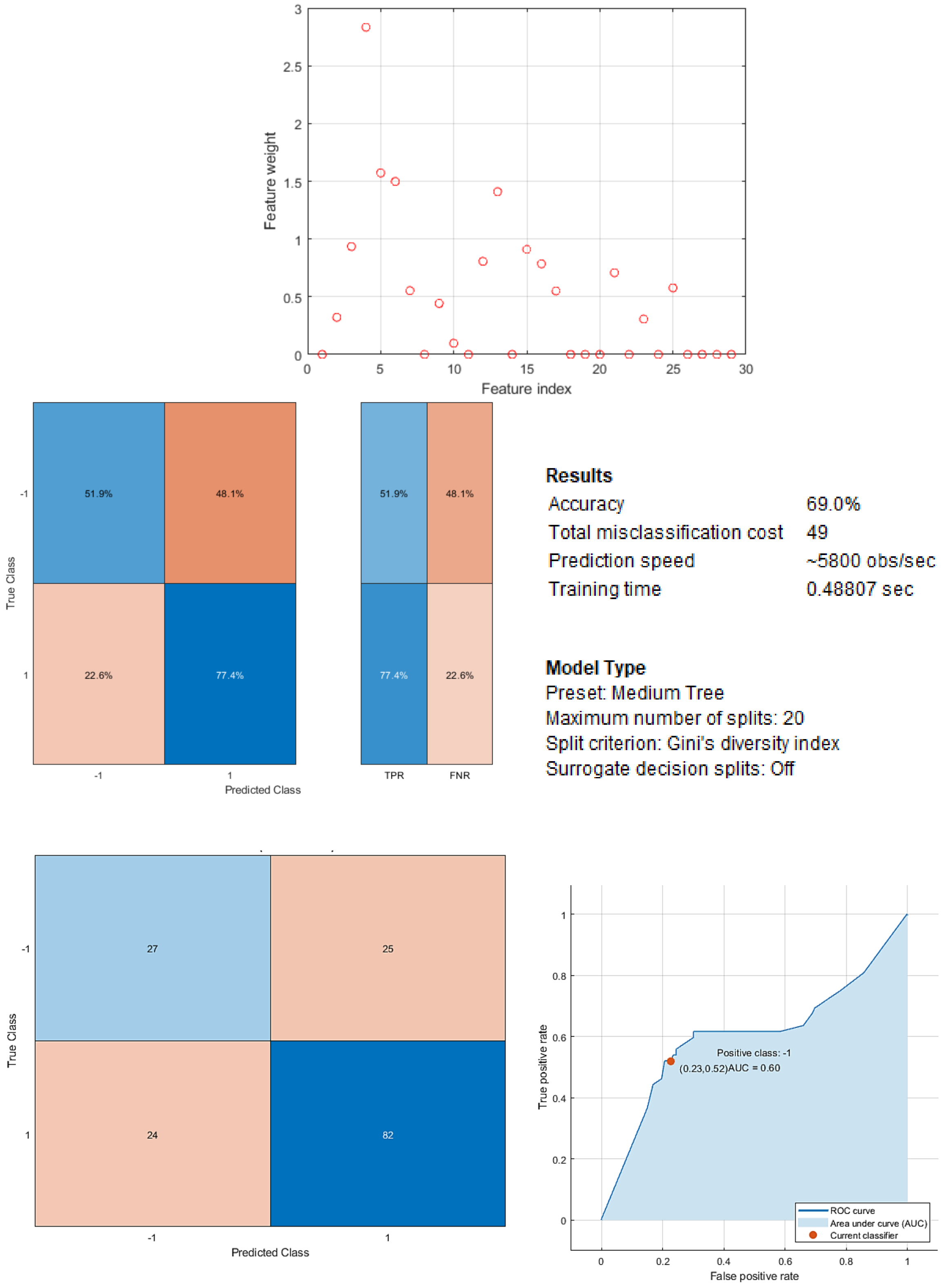

Model 3.2. Output for reduction or increase (binary) of musculoskeletal disorders in the neck with selected features According to the diagram below, Follow-up 6 month. Medium tree model.
